# Different Immunological Phenotypes Associated with Preserved CD4+ T Cell Counts in HIV-Infected Controllers and Viremic Long Term Non-Progressors

**DOI:** 10.1371/journal.pone.0063744

**Published:** 2013-05-16

**Authors:** Julie Christine Gaardbo, Hans J. Hartling, Andreas Ronit, Kristina Thorsteinsson, Hans Ole Madsen, Karoline Springborg, Lise Mette Rahbek Gjerdrum, Carsten Birch, Matthew Laye, Henrik Ullum, Åse Bengaard Andersen, Susanne Dam Nielsen

**Affiliations:** 1 Viro-immunology Research Group, Department of Infectious Diseases, Rigshospitalet, University Hospital of Copenhagen, Copenhagen, Denmark; 2 Department of Clinical Immunology, Rigshospitalet, University Hospital of Copenhagen, Copenhagen, Denmark; 3 Department of Infectious Diseases, Hvidovre Hospital, University Hospital of Copenhagen, Copenhagen, Denmark; 4 Department of Oto-rhinolaryngology, Rigshospitalet, University Hospital of Copenhagen, Copenhagen, Denmark; 5 Department of Pathology, Rigshospitalet, University Hospital of Copenhagen, Copenhagen, Denmark; 6 Center of Inflammation and Metabolism, Rigshospitalet, University Hospital of Copenhagen, Copenhagen, Denmark; 7 Department of Infectious Diseases, Odense Hospital, University of Southern Denmark, Odense, Denmark; Massachusetts General Hospital, United States of America

## Abstract

**Background:**

HIV-infected controllers control viral replication and maintain normal CD4+ T cell counts. Long Term Non-Progressors (LTNP) also maintain normal CD4+ T cell counts, but have on-going viral replication. We hypothesized that different immunological mechanisms are responsible for preserved CD4+ T cell counts in controllers and LTNP.

**Methods:**

25 HIV-infected controllers and 14 LTNP were included in this cross-sectional study. For comparison, 25 progressors and 34 healthy controls were included. Production and destruction of T cells were addressed by determination of T cell receptor excision circles (TREC), recent thymic emigrants, naïve cells, immune activation, senescence and apoptosis. Furthermore, telomere length was determined, and the amount of lymphoid tissue in tonsil biopsies was quantified.

**Results:**

Controllers presented with partly preserved thymic output, preserved expression of the IL-7 receptor and IL-7 receptor density, and lower levels of destruction of cells than progressors resembling HIV-negative healthy controls. In contrast, LTNP appeared much like progressors, and different from controllers in immune activation, senescence, and apoptosis. Interestingly, CD8+ RTE, TREC and telomere length were partly preserved. Finally, both controllers and LTNP displayed decreased amounts of lymphoid tissue compared to healthy controls.

**Conclusions:**

Controllers presented with an immunological profile different from LTNP. While controllers resembled healthy controls, LTNP were similar to progressors, suggesting different immunological mechanisms to be responsible for preserved CD4+ T cell counts in LTNP and controllers. However, both controllers and LTNP presented with reduced amounts of lymphoid tissue despite preserved CD4+ T cell counts, indicating HIV to cause damage even in non-progressors.

## Introduction

A minority of individuals infected with human immunodeficiency virus (HIV) do not progress to acquired immunodeficiency syndrome even in the absence of treatment. Some of these patients are able to control viral replication and maintain stable CD4+ T cell counts, and these patients are referred to as elite controllers (EC, <50 copies/mL) or viremic controllers (VC, 50–2,000 copies/mL), respectively. Another group of non-progressing patients also maintain normal CD4+ T cell counts for several years, but in contrast to controllers these patients have on-going viral replication. This group of patients is therefore termed Long Term Non-Progressors (LTNP) [1]. The mechanism behind the lack of disease progression in LTNP is largely unknown.

Importantly, both LTNP and controllers are infected with replicant-competent virus [2] suggesting the host immune system to be involved in maintaining normal CD4+ T cell counts. Initiation of combination antiretroviral therapy (cART) in HIV-infected patients reverts or partially reverts the immunological dysfunctions described in untreated HIV-infection, suggesting viral replication to be a leading cause of immunological dysfunction. Thus, in controllers the mechanisms leading to viral control may be responsible for the preserved CD4+ T cell counts as well. In contrast, viral control cannot explain the non-progression in viremic LTNP prompting the question how LTNP are able to maintain normal CD4+ T cell counts despite ongoing viral replication. LTNP can be seen as a human pendant to the natural hosts of simian immunodeficiency virus (SIV), sooty mangabeys and African green monkeys, who do not progress despite a high viral load. These monkeys do not show any signs of increased immune activation or T cell turnover [3], [4] which is therefore expected in LTNP as well.

The CD4+ T cell count is the result of production, destruction and traffic between blood and lymphatic tissue, and when destruction exceeds production the CD4+ T cell count decreases. Previous studies have shown differences in T cell subsets between LTNP *or* controllers and progressors. However, to our knowledge comparison of features of the adaptive immune system in LTNP and controllers has not been examined.

This study aimed to investigate production, destruction and distribution of T cells in HIV-infected controllers, LTNP and progressors in order to identify differences which could generate hypotheses for mechanisms leading to lack of disease progression in LTNP and controllers. Production was addressed by measurement of T cell receptor excision circles (TREC), recent thymic emigrants (RTE) and naïve cells. Immune homeostasis was addressed by expression of the IL-7 receptor (IL-7R) frequency and density Destruction was addressed by measuring immune activation, senescence and apoptosis. Distribution of cells was assessed by quantifying the amount of lymphoid tissue in tonsil tissue. As the length of the telomeres is a result of both production and destruction, telomere length were examined as well.

## Methods

### Ethics Statement

Informed consent was obtained in writing and verbally from all participants. The study was performed in accordance with the ethical guidelines of the 1975 Declaration of Helsinki and approved by the Local Ethical Committee (H-2-2009-089) and the Danish Data Protection Agency.

### Study Design

A total of 64 HIV-infected patients and 34 healthy controls were included in this cross-sectional study. The patients were divided into three groups: (1) 14 LTNP with HIV-infection for a minimum of ten years, HIV RNA >5,000 copies/mL and no decay in CD4+ T cell counts for a minimum of 2 consecutive years prior to inclusion, (2) 25 controllers including 5 EC and 20 VC with HIV-infection for a minimum of 2 years and HIV RNA always <50 or 2,000 copies/mL, respectively, and (3) 25 progressors with a minimum rate of CD4+ T cell decay of 50 cells/µL/year for a mimimum of two consecutive years prior to inclusion, and HIV RNA >5,000 copies/mL. All patients were to have CD4+ T cell counts within the normal range (reference interval 390–1200 cells/µL). Separate analyses were made for EC and VC. However, no differences were found in any aspects between EC and VC, and the two groups were therefore combined and are referred to as controllers. For comparison, 34 HIV-negative healthy controls matched for age, gender and ethnicity were included (Table 1). Exclusion criteria were co-infection with hepatitis B or C, other acute or chronic ongoing infections, malignant disease, immunosuppressive treatment, and pregnancy. All patients were enrolled from Department of Infectious Diseases, Rigshospitalet or Hvidovre Hospital, University of Copenhagen. Healthy controls were recruited among hospital staff.

**Table 1:** Clinical characteristics of the full study population and of those having a tonsil biopsy made.

Total study population	HIV−	Controllers	LTNP	Progressors
**Sex (males/total)**	**30/34**	**16/25**	**14/14**	**24/25**
**Age, years**	**43** (40–53)	**41** (35–51)	**45** (42–48)	**40** (37–45)
**CD4+ T cell count (cells/µL)**	**729** (613–900)	**660** (460–935)	**530** (480–610)	**525** (475–630)
**CD8+ T cell count (cells/µL)**	**369** (245–588)	**870** (659–1180)	**1200** (860–1600)	**1250** (932–1800)
**HIV RNA (log10 copies/mL)**	–	**3.14** (2.13–3.45)	**4.59** (4.34–4.81)	**4.60** (4.24–5.06)
**Infection duration (years)**	–	**7** (4–11)	**16** (10–20)	**3** (2–5)
**Patients having tonsil biopsies made**	**HIV−**	**Controllers**	**LTNP**	**Progressors**
**Sex (males/total)**	**8/9**	**7/9**	**5/5**	**7/7**
**Age (years)**	**41** (28–51)	**36** (28–42)	**44** (43–46)	**41** (27–48)
**CD4+ T cell count (cells/µL)**	**900** (725–1100)	**550** (470–915)	**500** (455–645)	**520** (480–650)
**CD8+ T cell count (cells/µL)**	**574** (338–697)	**830** (665–1000)	**1200** (740–2550)	**1200** (823–1300)
**HIV RNA (log10 copies/mL)**	–	**3.15** (2.57–3.23)	**4.76** (4.13–4.86)	**4.69** (4.34–5.0)
**Infection duration (years)**	–	**7** (4–15)	**13** (11–24)	**2** (2–6)

Data are given as median and (interquartile) ranges. HIV− = healthy controls, LTNP = Long Term Non Progressors.

### Blood Analysis

Ethylenediamine tetraacetic acid (EDTA) stabilised blood was used to obtain a full blood count and for flow cytometry. Plasma HIV RNA was measured with a polymerase chain reaction (PCR) quantitative kit (COBAS®AmpliPrep/COBAS®TaqMan® 48 System; Roche, Basel, Switzerland) according to the manufacturer’s instructions. Heparinised blood was used to isolate peripheral blood mononuclear cells (PBMC) by means of density gradient centrifugation. PBMC were frozen and later used for CD4+ and CD8+ T cell isolation.

### Flow Cytometry

**Figure 1:**
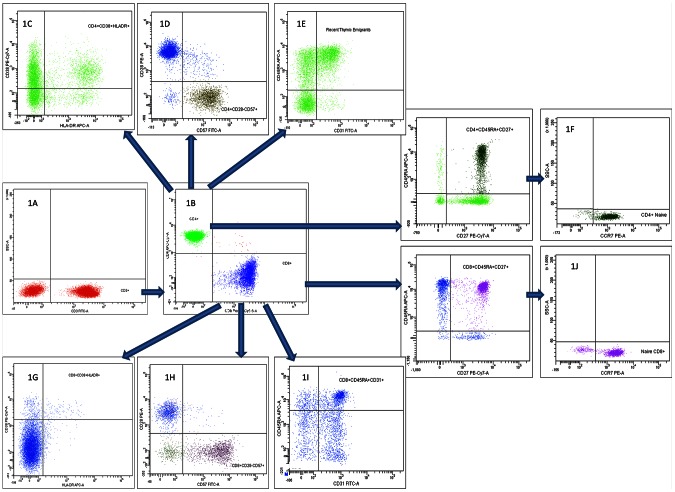
Flow cytometric gating strategy on CD4+ and CD8+ T cells and their subpopulations. First, CD3+T cells were identified (1A), and gating on CD4+ and CD8+ T cells were performed (1B). Second, CD4+ and CD8+ T cells subpopulations were identified as activated, senescent, recent thymic emigrants and naïve CD4+ T cells (1C, 1D, 1E and 1F, respectively), and activated, senescent, recent thymic emigrants and naïve CD8+ T cells (1G, 1H, 1I and 1J, respectively).

CD3 was used in combination with CD4 and CD8 to identify CD4+ and CD8+ T cells, respectively. Naive (CD27+CD45RA+CCR7+), RTE (CD45RA+CD31+), activated (CD38+HLA-DR+) and apoptotic (CD28–CD95+) and senescent (CD28–CD57+) CD4+ and CD8+ T cells were determined. Furthermore, IL-7R-expression (CD4+CD127+) was determined. Gating strategy is shown in fig. 1. In brief, 100 µL of EDTA blood was incubated with fluorescent dye–conjugated monoclonal antibodies according to the manufacturer’s instructions. Erythrocytes were lysed with Lysing Solution (Becton Dickinson, BD, NJ, USA), and samples were resuspended in Facs flow (BD).

Monoclonal antibodies used to determine lymphocyte subsets were isotype control IgG1/IgG2a Phycoerythrin (PE), IgG1 peridinin chlorophyll proteins-cyanine (PerCP-Cy5.5), IgG1/IgM fluorescein isothiocyanate (FITC), IgG1/IgG2b *Allophycocyanin (APC), IgG1 PE-Cy7, IgG1 APC-H7, CCR7-PE, CD28-PE, CD8-PerCP-Cy5.5, CD3-FITC, CD57-FITC, CD31-FITC, CD127-FITC, CD95-APC, HLA-DR-APC, CD45RA-APC, CD38-PE-Cy7 and CD4-APC-H7,* all purchased from BD. Six-colour acquisition was performed using a FACS Canto, and data were processed using FACS Diva software (BD). For each sample a minimum of 50,000 cells were acquired.

### Enrichment of CD4+ and CD8+ T Cells for DNA Isolation

Frozen PBMC were carefully thawed and separated into CD4+ T cells and CD8 T enriched PBMC using a magnetic cell separator (MACS) as described previously [5]. Briefly, PBMC were washed in phosphate-buffered saline (PBS, Sigma, MO, USA) supplemented with fetal calf serum (FCS, Sigma). Cells were incubated with CD4 microbeads (Miltenyl Biotech, Cologne, Germany) and passed through a 30 µm pre-separation filter (Miltenyl Biotec). Separation was carried out on MACS® separation columns (Miltenyl Biotec). The separation process was repeated for the CD4+ T cell depleted fraction. After purification, cells were stained with CD3, CD4 and CD8. CD4+ T cells were determined as CD3+ CD4+, CD8+ T cells were determined as CD3+ CD8+, and purity was measured using flow cytometry. The CD4+ T cell content in the purified CD4+ T cells was always >90%. The CD8+ T cell content in the CD8+ T cell enriched fraction was mean 77%.

### Isolation of DNA

DNA was used for determination of TREC and telomere length. DNA was isolated from purified CD4+ T cells and from CD8+ enriched PBMC using the Promega Wizard SV kit (Promega, Madison, Wisconsin, USA) according to manufacturer’s instructions.

### TREC

Quantification of signal-joint TREC was done by real-time quantitative PCR with the 5-nuclease (TaqMan) assay. TaqMan probes were dual-labelled ZEN-probes (Integrated DNA Technology, Leuven, Belgium). Samples were performed on a Stratagene 3005P realtime PCR-platform, analyzed in duplicates and averaged. The SJ TREC RQ-PCR system was changed as suggested [6].

### Telomere Length

Telomere length was measured as previously described [7]. Briefly, DNA was quantified using a Nanodrop ND 1000 (Saveen biotech ApS, Aarhus, Denmark). DNA was amplified in reaction mixture containing SYBR green master mix (Applied Biosystems, Foster City, CA,) and primers either targeting the telomere or the single copy gene 36B4. PCR conditions for telomere amplification were 40 cycles of 95°C for 15 s, 54°C for 2 min and for 36B4 40 cycles of 95°C for 15 s and 58°C for 1 min. Standard curves and dissociation curves for each primer set were done. Relative telomere to 36B4 ratios were calculated using the ΔΔCt method (User Bulletin No. 2, ABI PRISM 7700 Sequence Detection System). 36B4 did not differ between groups. Data are presented as a percentage relative to healthy controls and normalized to 36B4. Thus, mean values in healthy controls equals 1, while the median may differ from 1.

### Tonsil Biopsies

Along with blood samples tonsil biopsies were performed in those participants with preserved tonsils willing to donate a biopsy (n = 30). Biopsies were done by applying local anaesthesia followed by biopsy from the one tonsil easiest accessible with Weils forceps and knife. Haemostasis was secured by compression and ice application. All biopsies were performed by the same experienced oto-rhinolaryngologist (KS).

**Figure 2:**
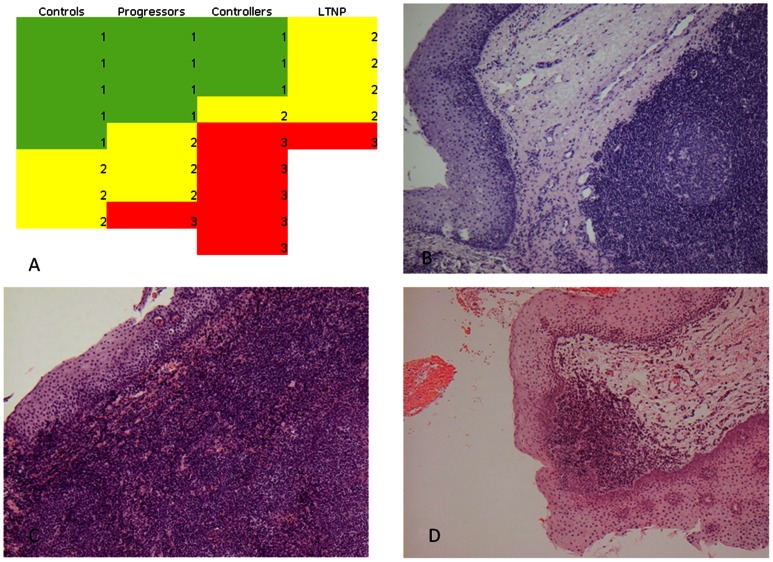
Data on lymphoid tissue in tonsil biopsies. In 8 healthy controls (HIV-), 8 progressors, 9 controllers and 5 long term non-progressors (LTNP) tonsil biopsies were performed. In both controllers and LTNP more patients appeared to have a disrupted lymphoid architecture with reduced amounts of lymphoid tissue compared to healthy controls (p = 0.0263 and p = 0.0570, respectively), while progressors were comparable to healthy controls. Fig. 2A shows the lymphoid score. Each box represents one biopsy. Histological characteristics of the lymphoid tissue were scored as stage I (no lymphocyte depletion with prominent germinal centres, green), stage II (partial lymphocyte depletion with >10% lymphocytes, with or without small follicles, no germinal centres, yellow) or stage III (lymphocyte depletion with <10% lymphocytes, no germinal centres or follicles, red). Fig. 2B, 2C and 2D show representative pictures of stage I, II and III, respectively.

Biopsies were fixed in neutral buffered formalin (4%) for at least overnight, followed by paraffin embedding. Serial 2 µm sections were cut and processed for hematoxylin eosin staining. Morphological evaluation of the whole biopsy was performed as suggested by Mozos [8] blinded to clinical data. Histological characteristics of the lymphoid tissue were scored as stage I (no lymphocyte depletion with prominent germinal centres), stage II (partial lymphocyte depletion with >10% lymphocytes, with or without small follicles, no germinal centres) or stage III (lymphocyte depletion with <10% lymphocytes, no germinal centres or follicles), (Fig. 2).

### Statistical Analyses

Differences between groups were analysed using Kruskal-Wallis followed by the Mann-Whitney U test to compare ranks between groups. Associations were examined by Spearman rank order correlations. Two-tailed p-values less than 0.05 were considered significant. Results are given as median percentages (%) and inter quartile ranges (IQR). Data were analyzed using Graphpad Prism 5.0, (GraphPad Software, La Jolla, California, USA).

## Results

### Production and Homeostasis of CD4+ T Cells

**Figure 3:**
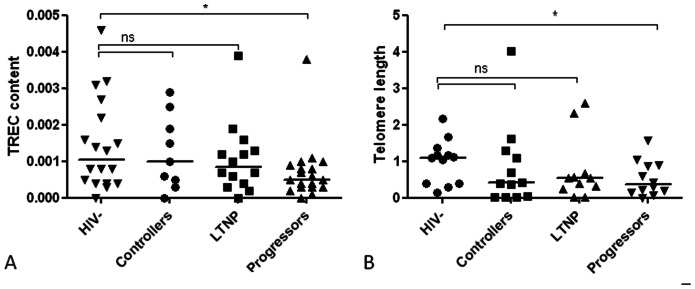
T cell receptor excision circles (TREC) and telomere length. Fig. 3A shows median (interquartile range) TREC content in DNA in healthy controls (HIV−, n = 18), controllers (n = 9), long term non-progressors (LTNP, n = 14) and progressors (n = 19) were 0.0011 (0.0004–0.0023), 0.001 (0.0004–0.0022) 0.0009 (0.0004−0.0014) and 0.0005 (0.0003−0.0009), respectively. Fig. 3B shows median (interquartile range) telomere length in healthy controls (n = 12), controllers (n = 9), LTNP (n = 11) and progressors (n = 12) were 1.09 (0.40−1.32), 0.39 (0.03−1.36), 0.55 (0.24−0.66) and 0.36 (0.15−0.89), respectively. Values are given relative to healthy controls. Both TREC and telomere length were partly preserved in controllers and LTNP compared to healthy controls (all p values >0.05), while progressors displayed both reduced TREC content and telomere length (p = 0.046 and p = 0.035, respectively). *indicates p values <0.05, ns indicates not significant.

Production of naive T cells in the thymus is crucial for the maintenance of T cell homeostasis. We hypothesised that markers of increased production including TREC content, CD4 RTE, naive T cells and expression of the IL-7R on CD4+ T cells differed between progressors and non-progressors. Differences between groups were not found in RTE or in percentages of naive CD4+ T cells, (Table 2). However, TREC content in LTNP and controllers were partly preserved and not significantly different from healthy controls, while progressors displayed lower TREC content (p = 0.046, Fig. 3A). Also, a tendency towards higher TREC in controllers compared to progressors were found (p = 0.086, Fig. 3A). Highly significant association was found between naive CD4 T cells and CD4+ RTE (all p-values <0.0001). No associations were found between naive CD4+ T cells or CD4+ RTE and TREC (data not shown). IL-7R expression in controllers was preserved with a frequency similar to healthy controls, while it was decreased in LTNP and progressors compared to both healthy controls (p = 0.017 and 0.026, respectively) and to controllers (p = 0.018 and 0.011 respectively). Furthermore, IL-7R density measured as mean flouroscence intensity (MFI, Table 2) was elevated in controllers compared to progressors and LTNP (p = 0.013 and 0.019, respectively).

**Table 2:** Data on production and destruction of CD4+ and CD8+ T cells.

	HIV-	Controllers	LTNP	Progressors	P value
	N = 34	N = 25	N = 14	N = 25	
**Naïve CD4+ T cells**	**45.5%**	**41.7%**	**50.4%**	**43.7%**	>0.05
(% of CD4+ T cells)	(38.9–55.0)	(33.8–51.9)	(35.7–54.4)	(36.6–52.4)	
**CD4+ RTE**	**23.3%**	**20.6%**	**24.8%**	**25.6%**	>0.05
(% of CD4+ T cells)	(15.2–31.0)	(16.3–29.9)	(20.2–27.8)	(17.2–30.6)	
**IL-7R/CD127**	**81.9%**	**86.1%**	**78.1%**	**69.4%**	controllers vs. HIV− >0.05
(% of CD4+ T cells expressing IL-7R)	(80.4–86.1)	(76.4–88.9)	(65.9–82.8)	(60.3–86.5)	LTNP vs. HIV– = 0.017 controllers vs. LTNP = 0.018 PR vs. HIV– = 0.026
**IL-7R/CD127**	**233**	**261**	**223**	**203**	controllers vs. LTNP = 0.019
(MFI)	(221–261)	(226–281)	(188–251)	(178–263)	controllers vs. PR = 0.013 controllers vs. HIV− = 0.049
**Activated CD4+ T cells**	**0.9%**	**1.5%**	**3.4%**	**4.2%**	LTNP vs. controllers = 0.007
(% of CD4+ T cells)	(0.8–1.8)	(0.7–2.9)	(2.3–3.9)	(2.6–7.6)	controllers vs. PR = 0.0002 LTNP vs. PR >0.05
**Apoptotic CD4+ T cells**	**0.9%**	**6.5%**	**6.1%**	**8.5%**	All HIV+ vs. HIV− <0.01
(% of CD4+ T cells)	(0.3–3.8)	(2.3–11.8)	(2.7–14.4)	(2.7–18.2)	
**Senescent CD4+ T cells**	**0.9%**	**3.3%**	**6.6%**	**7.7%**	All HIV+ vs. HIV− <0.01
(% of CD4+ T cells)	(0.1–3.4)	(1.4–9.4)	(3.4–13.3)	(2.3–16.4)	
**Naïve CD8+ T cells**	**35.3%**	**18.0%**	**12.5%**	**8.0%**	All HIV+ vs. HIV− <0.001
(% of CD8+ T cells)	(22.1–49.9)	(12.7–31.0)	(5.0–18.0)	(5.0–13.1)	LTNP vs. controllers = 0.073 controllers vs. PR = 0.0004 LTNP vs. PR >0.05
**CD8+ RTE**	**38.5%**	**30.5%**	**22.2%**	**13.9%**	Controllers vs. PR <0.0001
(% of CD8+ T cells)	(30.7–58.7)	(20.8–38.8**)**	(21.1–25.6)	(11.0–21.3)	LTNP vs. PR = 0.01
					All HIV+ vs. HIV− <0.01
**Activated CD8+ T cells**	**3.1%**	**10.2%**	**21.7%**	**22.4%**	All HIV+ vs. HIV− <0.0001
(% of CD8+ T cells)	(1.8–4.6)	(4.3–24.0)	(15.3–26.1)	(16.1–34.4)	LTNP vs. controllers = 0.077 controllers vs. PR = 0.0057 LTNP vs. PR >0.05
**Apoptotic CD8+ T cells**	**25.1%**	**48.6%**	**59.6%**	**63.8%**	All HIV+ vs. HIV− <0.0001
(% of CD8+ T cells)	(15.1–40.0)	(39.1–63.5)	(49.2–71.1)	(48.4–70.3)	LTNP vs. controllers = 0.049 controllers vs. PR = 0.026 LTNP vs. PR >0.05
**Senescent CD8+ T cells**	**17.8%**	**29.5%**	**35.2%**	**34.5%**	All HIV+ vs. HIV− <0.05
(% of CD8+ T cells)	(11.1–33.8)	(23.3–40.3)	(27.2–38.7)	(29.1–45.8)	controllers vs. PR = 0.095

Data are given as median and (interquartile ranges) in percentages (%) of CD4+ or CD8+ T cells, respectively. LTNP: long term non-progressors, PR: progressors, HIV−: healthy controls, MFI: Mean Flouroscence Intensinty.

### Destruction of CD4+ T Cells

**Figure 4:**
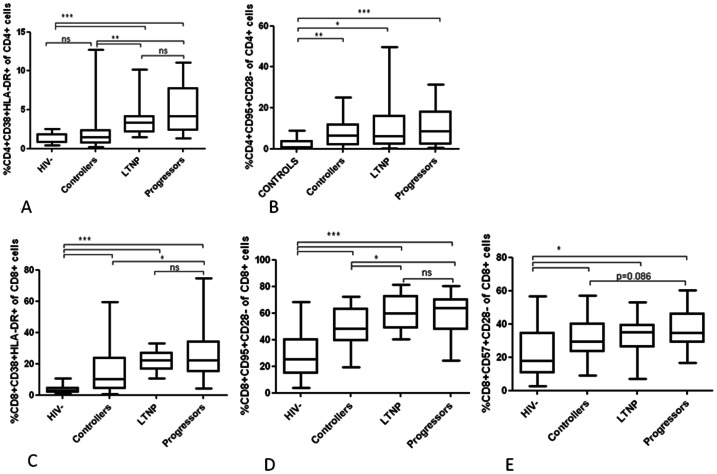
Activated, senescent and apoptotic T cells. Fig. 4A and 4B shows activated and apoptotic CD4+ T cells, respectively, while Fig. 4C,D and E shows activated, senescent and apoptotic CD8+ T cells, respectively, in healthy controls (HIV−, n = 34), controllers (n = 25), long-term non-progressors (n = 14) and progressors (n = 25). Values are presented as percentages (%) of CD4 and CD8+ T cells, respectively. In all cell populations HIV-infected patients displayed elevated percentages compared to healthy controls. Furthermore, in all cell populations controllers resembled healthy controls, while LTNP resembled progressors. *indicates p values <0.05, **indicates p values <0.001, ***indicates p values <0.0001, ns indicates not significant.

The rate of destruction of T cells may influence T cell homeostasis, and activated and apoptotic CD4+ T cells were expected to differ between progressors and non-progressors. Activated CD4+ T cells were elevated in LTNP and progressors compared to healthy controls (<0.0001 and <0.0001, Fig. 4A), while controllers did not significantly differ from controls (p = 0.120). Controllers presented with lower immune activation compared to LTNP and progressors (p = 0.007 and 0.0002), while there were no differences between LTNP and progressors. Frequencies of apopotic and senescent CD4+ T cells were elevated in all groups of HIV-infected patients compared to healthy controls (Fig. 4B and Table 2).

### Production of CD8+ T Cells

Frequencies of naive CD8+ T cells in all groups of HIV-infected individuals were lower compared to healthy controls (all p values <0.001), (Table 2). Controllers had higher levels of naive CD8+ T cells compared to progressors (p = 0.0004), while there were no difference between LTNP and progressors. Frequencies of CD8+ RTE were partly preserved in both LTNP and controllers. In contrast CD8+ RTE was reduced in progressors compared to healthy controls (Table 2).

### Destruction of CD8+ T Cells

Activated, immunological senescent and apoptotic CD8+ T cells were determined. Elevated frequencies of activated CD8+ T cells in controllers, LTNP and progressors compared to healthy controls were found (p values <0.0001, Fig. 4C). Frequencies in controllers were lower than in progressors (p = 0.006). No difference between LTNP and progressors was found. Similar findings regarding apoptotic CD8+ T cells were recorded with higher frequencies in all groups of HIV−infected patients (p values <0.0001, Fig. 4D). However, controllers had lower frequencies compared to LTNP and progressors (p = 0.049 and 0.025), while there was no difference between LTNP and progressors.

Frequencies of senescent CD8+ T cells in controllers, LTNP and progressors were elevated compared to healthy controls (p = 0.017, 0.028 and 0.0005, Fig. 4E). A tendency towards lower frequencies in controllers compared to progressors was found (p = 0.086), while there were no differences between LTNP compared to controllers and progressors.

### Telomere Length

To further address T cell homeostasis length of the telomeres were examined in CD8+ T cells. Telomere length was partly preserved in both controllers and LTNP with no significant difference from healthy controls, while progressors had reduced telomere length (p = 0.0350, Fig. 3B).

### Lymphoid Tissue

To examine the contribution of lymphoid tissue to the T cell homestasis, tonsil biopsies were performed. Disruption of the lymphoid tissue was expected in HIV-infected patients. A total of 30 patients and controls had a biopsy made from the tonsils. The study population having biopsies made was representative of the full study population (Table 1). In both LTNP and controllers, there were more individuals with disrupted architecture in the lymphoid tissue compared to healthy controls (p = 0.026 and p = 0.057, respectively, Fig.2). In contrast, progressors were similar to healthy controls (p = 0.550). There seemed to be a negative association between duration of HIV-infection and the amount of lymphoid tissue (data not shown).

### Impact of the Viral Load

To address the impact of the viral replication on T cell homeostasis, possible associations between HIV and the examined parameters were examined.

In LTNP and progressors positive associations were found between HIV RNA and immune activation in both the CD4+ and CD8+ T cell compartment. In controllers, a strong negative association was found between HIV RNA and TREC. Also, a positive association between HIV RNA and senescent and apoptotic CD8+ T cells was found in controllers. Results are presented in Table 3.

**Table 3:** Associations between HIVRNA and parameters examined in Long term Non-Progressros (LTNP), controllers and progressors.

HIVRNA vs.	Controllers	LTNP	Progressors
	P-value (r-value)	P-value (r-value)	P-value (r-value)
**Naive CD4+ T cells**	**0.0232** (0.471)	ns	ns
**Naive CD8+ T cells**	ns	ns	**0.0470** (−0.401)
**CD4+ RTE**	**0.0212** (0.458)	ns	ns
**TREC**	**0.0211** (−0.829)	ns	ns
**Activated CD4+ T cells**	ns	**0.0044** (0.710)	**0.0005** (0.642)
**Activated CD8+ T cells**	ns	**0.0257** (0.613)	**0.0222** (0.455)
**Senescent CD8+ T cells**	**0.0400** (0.422)	ns	ns
**Apoptotic CD8+ T cells**	**0.0051** (0.552)	ns	ns

Cell populations are given as percentages of CD4+ or CD8+ T cells, respectively. Only significant results are given. Ns indicates not significant.

To further address the influence of the viral replication patients in the three groups were divided into subgroups with high vs. low viral replication by determining the median, and comparisons were done. In the LTNP group those patients with the highest level of viremia presented with higher frequency of activated CD4+ T cells compared to those with lower viral replication (p = 0.026). The progressor group with the highest level of viral replication displayed higher frequency of activated CD4+ T cells (p = 0.002) and apoptotic CD8+ T cells (p = 0.047), and lower frequnecy of naive CD8+ T cells (p = 0.013) compared to the group with lower viral replication. In the controller group, those with the highest level of viral replication displayed lower frequency of naive CD4+ T cells (p = 0.031), higher frequency of apoptotic CD8+ T cells (p = 0.030), shorter telomere length (p = 0.037) and lower TREC (p = 0.034) compared to those patients in the group with low viral replication.

## Discussion

This study was designed to identify possible immunological factors of importance for non-progression in HIV-infected individuals. As hypothesized, controllers presented with a less dysfunctional immune system than progressors in both production and destruction of cells and partly preserved telomere length. Surprisingly, LTNP appeared very similar to progressors and different from controllers, although a partly preserved thymic output and telomere length was found. In contrast, both controllers and LTNP displayed decreased amounts of lymphoid tissue despite preserved CD4+ T cell counts.

LTNP and controllers are rare populations of HIV-infected patients characterized by non-progression with and without viral replication, respectively. However, definitions suffer from lack of consensus complicating comparisons across studies. In particular, LTNP are often defined without including the viral load. Thus, some LTNP may fulfill controller-criteria with low viral replication [9]–[12]. This may explain why most studies have evaluated either LTNP *or* controllers. In this study, LTNP were defined as HIV-infected individuals with no CD4+ T cell decay two years prior to inclusion and CD4+ T cell counts within the reference interval for >10 years without cART and viremia >5,000 copies/mL, as even 7 years of HIV-infection with stable CD4+ T cell counts does not seem to distinguish between LTNP and progressors [13]. Controllers were defined as HIV-infected individuals with stable CD4+ T cell counts for a minimum of 2 years and viremia never >2,000 copies/mL.

HIV leads to a disruption in the frequency and function of naïve CD4+ T cells in blood and lymphoid tissue [14]–[16]. Lack of disease progression may therefore be associated with preserved production of CD4+ T cells, and preserved TREC was found in controllers compared to progressors. Also, partly preserved CD8+ RTE were found in controllers and LTNP, but reduced in progressors, indicating partly preserved thymic output in both groups. In contrast, frequencies of naïve CD4+ T cells and CD4+ RTE were similar in controllers and progressors. In fact, these parameters were similar in all groups of HIV-infected patients and comparable to healthy controls. These findings are consistent with earlier studies, although decreased proportions of naïve CD4+ T cells has been described in elite controllers [9], [17]–[20]. The similar level of thymic output in our study may reflect that all examined individuals had CD4+ T cell counts within the reference interval and a yet functional thymus able to meet the demands of production of CD4+ T cells. IL-7 is crucial in the T cell homeostasis, and the IL-7 responsiveness is determined largely by the presence or absence of the IL-7R [21]. A negative association between IL-7 and CD4+ T cell count is described, and HIV-infected patients show high levels of IL-7 and reduced levels of IL-7R compared to healthy controls [22]. A preserved frequency of IL-7Rexpressing cells as well as high receptor density was found in controllers. This is in line with earlier findings of lower levels of IL-7R in progressors compared to a group of LTNP where some fulfilled controller-criteria [9]. This suggests low viral replication to lead to low rate of infection of CD4+ T cells. Thereby, the level of IL-7 does not rise and the IL-7R expression remains high.

Immune activation is a well-known predictor of disease progression in HIV-infection [23], [24] accompanied by immunological senescence and apoptosis [25], [26]. In controllers, lower immune activation senescence and apoptosis were found in both the CD4+ and CD8+ cell compartement in accordance with multiple previous studies [10], [19], [27]–[32]. Due to the predictive nature of immune activation it seems reasonable to consider the low immune activation in controllers to be a contributor to lack of progression. Another way to assess cell turnover is to determine telomere length. The functional and phenotypic changes of senescent CD8+ T cells are accompanied by erosion of telomere length and fading telomerase expression [33]. Telomeres have been shown to decrease with each replication of DNA [34], and shortened telomeres are reported in CD8+ T cells from HIV-infected individuals [35]. Partly preserved telomere length in CD8+ T cells comparable to healthy controls was found in controllers and LTNP, possibly reflecting a different homeostasis in non-progressors compared to progressors.

In contrast to controllers, LTNP were found to be similar to progressors with no differences found in immune activation, senescence and apoptosis. Thus, the phenomenon of LTNP does not appear to be comparable to the non-progressing natural hosts of SIV. Immune activation in LTNP is poorly described with conflicting results [36], [37], while senescence and apoptosis is previously undescribed. Likewise, IL-7R was decreased in both progressors and LTNP, suggesting IL-7 homeostasis not to be a key factor in non-progression.

Unlike other parameters examined, partly preserved TREC content and CD8+ RTE were found in LTNP. Of note, LTNP in this study had median duration of infection of sixteen years, suggesting a large thymic capacity to be involved in non-progression in LTNP. This is supported by earlier findings of thymic output to predict disease progression [12], [38], [39]. Furthermore, a transient nature of LTNP has been suggested as most LTNP seem to progress eventually [20], [40], [41], possibly explained by declining thymic capacity with age [15]. Also, partly preserved telomere length was found in LTNP highlighting these patients to have a more beneficial balance between production and destruction than progressors. Finally, one could speculate whether the cross-sectional design and the inclusion criteria of the study prevent us from determining if the LTNP have actually been different from progressors earlier in the infection at a time where their LTNP-status was not yet evident. Also, this study was limited by lack of mechanistic parameters. Indeed, it would be interesting to evaluate if any of the examined flow cytometric parameters are predictive of the clinical phenotype. This was recently proposed in a statistical model of aviremic HIV-infected individuals including >300 individuals [42]. However, a larger cohort than in the present study is necessary to develop such a model.

Controllers and LTNP displayed surprisingly many differences, suggesting different immunological mechanisms to be responsible for non-progression. This is supported by one of the only previous studies comparing LTNP and controllers showing similar findings in natural killer cells from LTNP and progressors different from controllers and healthy controls [37]. Additionally, a recent study of non-progressing as well as progressing controllers revealed differences in thymic output between these two groups [18]. The fact that some controllers progress despite undetectable viral replication supports the idea that CD4+ T cell homeostasis and control of viral replication are distinct but co-inciding processes. In this context, the impact of the viral replication should be considered. In this study, positive associations were found between immune activation and HIV RNA in LTNP and progressors, and those patients with the highest viremia presented with the highest immune activation, indicating the viral replication to influence on immune activation. In controllers, a strong negative association was found between HIV RNA and TREC, and those patients with the highest viremia also displayed significantly lower TREC levels, suggesting in the controllers even very low levels of viremia to have a negative influence on thymic output.

Finally, while progressors were not different from healthy controls, both controllers and LTNP seemed to have reduced amounts of lymphoid tissue in their tonsils in line with earlier findings of HIV damaging lymphoid tissue [1]. Recently damaged lymphoid tissue in the gut was shown in HIV-infected patients, including controllers [43]. The more preserved lymphoid tissue in progressors may, however, be explained a shorter period of infection. All together these findings are suggestive of an overall damaging effect of HIV on lymphoid tissue in progressors as well as non-proggressors. However, few data exist on lymphoid tissue in general and non-progressors have rarely been examined. One study reports non-progressors to have a more preserved structure of lymph nodes than progressors [44]. In our cohort, both controllers and LTNP had been HIV-infected for a longer time than the progressors, and a negative association between infection duration and the amount of lymphoid tissue was found. Altogether, these data suggest infection with HIV to have a major impact on lymphoid tissue despite the ability to preserve CD4+ T cell counts. Thus, great compensatory mechanisms for the damaging effect of HIV, possibly a large capacity to produce cells, may be an important factor in non-progression.

In conclusion, controllers presented with an immunological profile different from LTNP. While peripheral immunology in controllers resembled healthy controls, LTNP were similar to progressors. These data suggest that different immunological mechanisms are responsible for preserved CD4+ T cell counts in LTNP and controllers. Many factors including increased production and decreased destruction seem to be involved in non-progression in controllers, although the low viral replication may be partly a consequence rather than a reason for non-progression. In contrast, the mechanism for non-progression in LTNP remains unclear, although a large thymic capacity with the ability to continuously meet the demands for production of CD4+ T cells in LTNP may be of importance. Despite preserved CD4+ T cell counts in both controllers and LTNP, both patient groups have disrupted lymphoid architecture in tonsil biopsies indicating damage induced by HIV. Indeed, future prospective and mechanistic studies are needed to disclose the mechanisms behind non-progression in these patients.
